# Gender Variations in the Effects of Number of Organizational Memberships, Number of Social Networking Sites, and Grade-Point Average on Global Social Responsibility in Filipino University Students

**DOI:** 10.5964/ejop.v12i1.1040

**Published:** 2016-02-29

**Authors:** Romeo B. Lee, Rito V. Baring, Madelene A. Sta. Maria

**Affiliations:** a Behavioral Sciences Department, De La Salle University, Manila, Philippines; b Theology and Religious Education Department, De La Salle University, Manila, Philippines; c Psychology Department, De La Salle University, Manila, Philippines; Psychology Department, College of New Rochelle, New Rochelle, USA

**Keywords:** global social responsibility, organizational memberships, social networking sites, grade-point average, gender, Filipino university students

## Abstract

The study seeks to estimate gender variations in the direct effects of (a) number of organizational memberships, (b) number of social networking sites (SNS), and (c) grade-point average (GPA) on global social responsibility (GSR); and in the indirect effects of (a) and of (b) through (c) on GSR. Cross-sectional survey data were drawn from questionnaire interviews involving 3,173 Filipino university students. Based on a path model, the three factors were tested to determine their inter-relationships and their relationships with GSR. The direct and total effects of the exogenous factors on the dependent variable are statistically significantly robust. The indirect effects of organizational memberships on GSR through GPA are also statistically significant, but the indirect effects of SNS on GSR through GPA are marginal. Men and women significantly differ only in terms of the total effects of their organizational memberships on GSR. The lack of broad gender variations in the effects of SNS, organizational memberships and GPA on GSR may be linked to the relatively homogenous characteristics and experiences of the university students interviewed. There is a need for more path models to better understand the predictors of GSR in local students.

Young people, including those in the Philippines, are taught about global social responsibility (GSR) as part of their formal education in global citizenship (UNESCO, 2013). GSR is a crucial value to promote in this demographic sector, because the world’s burgeoning problems (e.g., climate change) demand both collective and cross-generational action. Several studies have examined GSR in young people, but their main focus has been on the effectiveness of educational activities on GSR (e.g., Bhandari & Belyavina, 2011) as well as on the statistical relationships of GSR with other global citizenship elements (e.g., Blake & Reysen, 2014). There is a sparse amount of attention bestowed to investigating the effects of factors at the individual level. Like other cultural values, GSR is likewise influenced by elements stemming from individual-based factors, such as one’s social activities and academic performance. This study discusses the effects of number of organizational memberships, number of social networking sites (SNS), and grade-point average (GPA) on GSR. These effects are hypothesized to differ according to gender, which is known to influence the learning of human values (Bowl, Tobias, Leahy, Ferguson, & Gage, 2012; Severiens & Dam, 1994). Studying these factors is important as it would determine potential pathways to effectively foster GSR in young people.

In their review of prominent theoretical and philosophical literature, Morais and Ogden (2011) define GSR in terms of *interdependence* and *social concern for others, the society and the environment.* According to them, these measures specifically indicate: 1) understanding global interconnectedness and personal responsibility; 2) knowing global issues and being emphatic of global justice and disparities; and 3) constructing an ethic of social service to help resolve social issues (Morais & Ogden, 2011). GSR clearly demands young people to situate themselves on a global scale and to embrace the call for service to the global community. The learning of the value may constitute a challenge, therefore, for many young people who are still in the process of forming their self-concept. Fortunately, individual-based factors that are beyond the structures of formal instruction have elements or values that could help enhance the movement of young people towards inculcating GSR. These factors require exploration.

Though their participation rate in youth organizations is low (European Commission, 2007), young people are continually encouraged to join organizations because of their developmental effects (Klindera & Menderwald, 2001). Studies have revealed that young people with an organizational membership or involvement tend to hold values and behaviors related to social responsibility (not necessarily at the global level). For example, students in the US who are members of an organization were found to possess a community value of citizenship. That is, these students “believed in a process whereby an individual and/or a group become responsibly connected to the community and to society through some activity; and recognized that members of communities are not independent, but interdependent and that individuals and groups have responsibility for the welfare of others” (Dugan & Komives, 2007, p. 10).

Moreover, US students having an organizational membership, relative to those without, were observed to have developed more humanitarian values and civic engagement (Hernandez, Hogan, Hathaway, & Lovell, 1999); and to have devoted longer hours to volunteering (Wolfe, 2014). These outcomes, which are likely influenced by the variety of activities that organizations commonly provide their members (e.g., organizing, networking and community service) (Hall, 2012), are amplified and deepened as young people gain more organizational memberships. Local social responsibility, which is commonly fostered by youth organizations, may serve as a springboard to fostering GSR in young people.

SNS play a very active role in connecting young people with others from countries all over the world. Using SNS represents a fundamental yet a significant practice among young people, including Filipino students. Facebook, the world’s most popular SNS, has 1.44 billion active users each month and 936 million users every day (Rushe, 2015); in the Philippines, it has 34.0 million active users (Kemp, 2012). In addition to Facebook, young people use other SNS; in fact, 71% of those in the US reported having more than one SNS (Lenhart, 2015). Apart from serving as channels for building social connections (Sheldon, Abad, & Hinsch, 2011) and inter-personal networks, SNS also constitute as main channels for acquiring and sharing news and information, including that on global and social justice issues (Ellison, Lampe, & Steinfield, 2009). These impacts are deepened as young people increase the number of their SNS. Having multiple SNS predicts global awareness. Young people with multiple SNS were reported to have felt knowledgeable about the world and interconnected with other people in the world (Lee, Baring, Sta. Maria, & Reysen, 2015). This global awareness is a precursor to the pro-social values of global citizenship (Reysen & Katzarska-Miller, 2013).

GPA is indicative of the quality of young people’s academic performance, but more importantly, of their hard work, conscientiousness, resilience and persistence. In large part, a high GPA is akin to gaining a mastery of and control over one’s academic life. As a less normative student characteristic than joining organizations and using SNS, a high GPA could potentially mediate rather than merely predict one’s inculcation of global values. A high GPA translates to a high self-esteem (Davidson, Metcalfe, Mueller, Molony, & Vodouris, 2012; Ruekberg, 2006). Since GPA is likewise associated with job and earning prospects (Oehrlein, 2009), a GPA-based self-esteem is by no means related only to school but also to broader life outcomes. Thus, young people with a high GPA are also likely to feel able, ready and open to accept and carry out global-level responsibilities. GPA is both an antecedent to global awareness and GSR. Young people with a high GPA have knowledge of the world and are concerned with global problems; value and respect cultural diversity and engage with other cultural groups; and most crucially, are willing to become global citizens (Lee et al., 2015, p. 2).

This study determines gender variations in the effects of number of organizational memberships, number of SNS, and GPA on GSR. There are gender differences in organizational memberships, SNS and GPA according to studies. For example, young women are more inclined to join organizations (Ferris, Oosterhoff, & Metzger, 2013) and to volunteer (Wolfe, 2014). There are more young women having multiple SNS (Jambulingam, Selvarajah, & Thuraisingam, 2014) whose main purpose for using social networks revolves around maintaining and deepening existing relationships, in contrast to young men who tend to continually establish new relationships in SNS (Mazman & Usluel, 2011). With respect to GPA, young men have lower grades compared to those earned by young women (Conger & Long, 2010).

As an overarching social structure (Risman, 2004), gender guides young men and women how to learn and act according to some prescribed characteristics. For instance, as a consequence of their gender-role socialization, young women, compared to men, are more relationship-oriented, emotional and caring (Tormey & Gleeson, 2012), and are more involved in acts of caring and support (Eagly, 2009). These gender characteristics are likely to define how young men and women would value and learn from organizational memberships, SNS and GPA, which would then impact on their GSR.

The purpose of the present study is to estimate gender variations in: 1) the direct effects of (a) number of organizational memberships, (b) number of SNS, and (c) GPA, on GSR; and 2) the indirect effects of (a) and (b) through (c) on GSR among Filipino university students. The structural path model estimated in this study is shown in Figure 1. It is hypothesized that number of organizational memberships and number of SNS would be positively associated with GSR in both genders. As a mediator, GPA would help enhance these positive associations.

**Figure 1:**
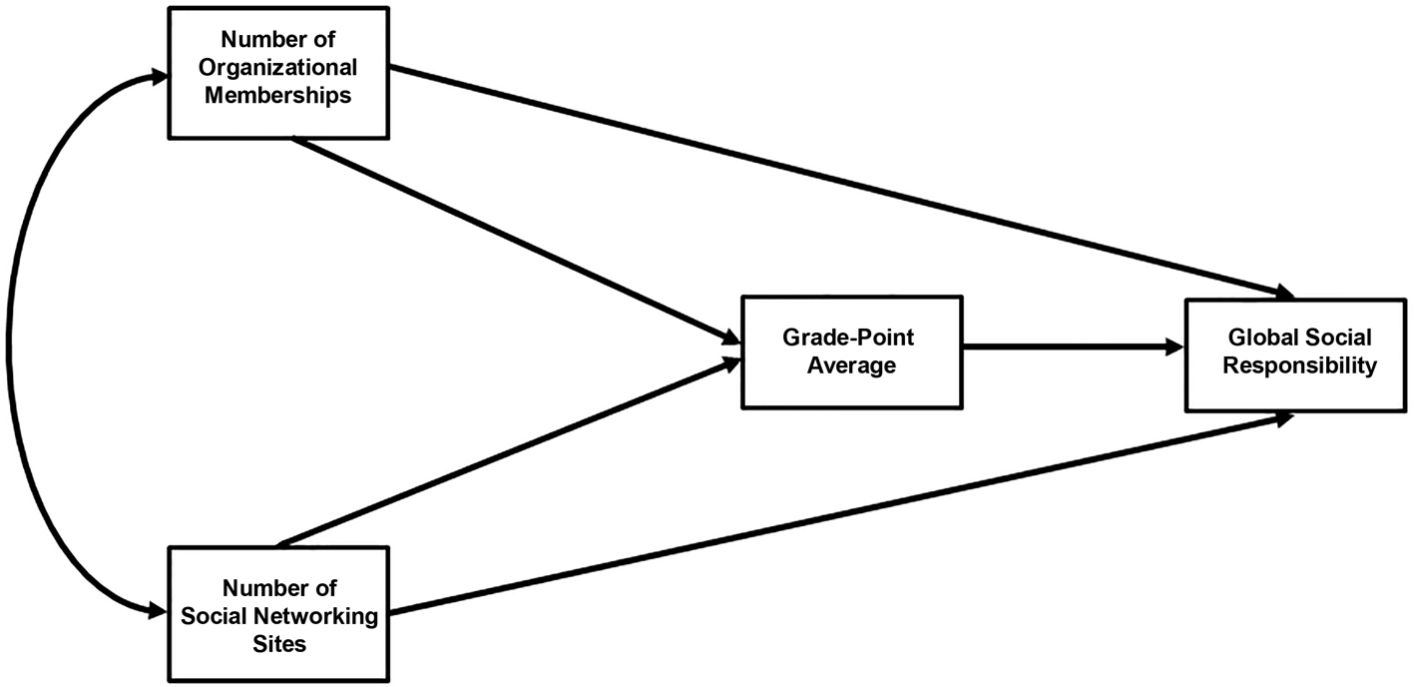
Structural path model.

## Methods

### Participants and Procedure

Using an anonymized self-administered questionnaire, the study interviewed 3,173 students enrolled at one university in Manila, Philippines. Of the sample, two-thirds were in first and second year levels. Three-fourths were enrolled in liberal arts, humanities and education, while the rest were completing engineering, natural sciences, business and economics, education, and computer courses.

The study presented in this report was part of a broader research that explored well-being, social and civic engagements, and relationships in young people. Ethical clearance was sought from the college research ethics review committee of the university. The questionnaire was administered in classrooms in the first quarter of the 90-minute regular classes. Those who consented to participate accomplished the questionnaire while attending an undergraduate general education class. Participants provided answers to questions that queried them about their number of organizational memberships, number of SNS, GPA and GSR. The average time to complete the questionnaire was 20 minutes.

### Materials

#### Number of Organizational Memberships

Participants were asked to indicate their membership in youth organizations inside and outside of their university.

#### Number of Social Networking Sites

For this item, participants indicated if they are members of Facebook (99.9%), Twitter (78%), LinkedIn (5%), Google Plus (31.3%), Tumblr (33%), and Instagram (66.3%). These six are the most popular sites among Filipino students. Based on reported memberships, an index of number of SNS was constructed.

#### Duration of Using Facebook

One item was used to measure the number of hours that participants spent using Facebook each day (1 = *≤5*, 2 = *6-10*, 3 = *11-15*, 4 = *16-20*, 5 = *>20*).

#### Grade-Point Average

Participants were asked to report their cumulative grade-point average. Their answers ranged from 0 *fail* to 4 *excellent.*

#### Global Social Responsibility

Twelve items on GSR, which were drawn from the Global Citizenship Scale of Reysen and Katzarska-Miller (2013), were asked of participants. These 12 items are pro-social values related to Intergroup Empathy (2 items), Valuing Diversity (2 items), Social Justice (2 items), Environmental Sustainability (2 items), Intergroup Helping (2 items), and Responsibility to Act (2 items). The internal consistency of the whole scale, as examined in the current data set, was very satisfactory (α = .88). The Social Responsibility dimension of the Global Citizenship scale, like the dimensions of global competence and global civic engagement, were empirically validated and found to be theoretically grounded (Morais & Ogden, 2011). In each item, participants checked a response between 1 = *strongly disagree* and 5 = *strongly agree*. Participants’ scores in all the mentioned items were added to form an index of GSR.

### Data Analysis

Path analysis, a form of structural equation modelling (Hox & Bechger, 2007), was used in this study. Based on the path model in Figure 1, factors were observed and tested to determine their relationships. Number of organizational memberships, number of SNS, and duration of using Facebook were the original exogenous predictors in the path model. GPA was the endogenous mediator predicted to influence the endogenous outcome, GSR. Path coefficient for the endogenous variable in the model was estimated. Standardized and unstandardized estimates were obtained for the direct effects of (a) number of organizational memberships, (b) number of SNS, and (c) GPA on GSR; and the indirect effects of (a) and (b) on GSR through GPA.

## Results

The descriptive characteristics of the sample are shown in Table 1. Mean GSR scores are statistically significantly lower for men than women (*M* = 48.2, *SE* = .16 versus *M* = 50.6, *SE* = .14, *t* = -2.43, *p* = .00). The GPA of men is similarly lower than that of women’s (*M* = 2.61, *SE* = .01 versus *M* = 2.92, *SE* = .01, *t* = -.32, *p* = .00). In addition, men in the study had fewer SNS memberships (*M* = 2.45, *SE* = .03 versus *M* = 3.16, *SE* = .02, *t* = -.71, *p* = .00); and fewer organizational memberships (*M* = .89, *SE* = .02 versus *M* = 1.00, *SE* = .01, *t* = -.12, *p* = .00). Both groups were almost identical with respect to duration of Facebook use.

**Table 1:** Descriptive Characteristics of Participants

Variables	Men (*N* = 1495, 47.1%)		Women (*N* = 1678, 52.9%)		Total (*N* = 3173)	
	*M*	*SE*	*M*	*SE*	*M*	*SE*
Global social responsibility	48.20***	.16	50.60***	.14	49.50	.11
Grade-point average	2.61***	.01	2.92***	.01	2.77	.01
Number of social networking sites	2.45***	.03	3.16***	.02	2.83	.02
Duration of using Facebook	1.29	.03	1.27	.03	1.28	.02
Number of organizational memberships	.89***	.02	1.00***	.01	.95	.01

*Note.* *SE* = Standard Error.

*p < .05. **p < .01. ***p < .001.

The correlations of the study variables are shown in Table 2. Except for one, all the exogenous variables are statistically significantly related with GSR. For men, the number of organizational memberships is shown to have the strongest positive correlation with GSR. For women, the variable with the strongest positive correlation with GSR is GPA. With its weak correlation with the endogenous variable, duration of using Facebook is excluded from the path analysis.

**Table 2:** Correlations Between Global Social Responsibility and Assessed Variables by Gender

Variables	1	2	3	4	5
Men					
1. Global social responsibility	1				
2. Grade-point average	.07*	1			
3. Number of social networking sites	.13**	.02	1		
4. Duration of using Facebook	-.01	-.03	.07*	1	
5. Number of organizational memberships	.14**	.12**	.20***	.03	1
Women					
1. Global social responsibility	1				
2. Grade-point average	.10**	1			
3. Number of social networking sites	.09*	-.06*	1		
4. Duration of using Facebook	-.01	-.00	.07*	1	
5. Number of organizational memberships	.07*	.15**	.06*	.00	1

*p < .05. **p < .01. ***p < .001.

The parameter estimates (unstandardized and standardized) for each path by gender are shown in Table 3. Based on the unstandardized coefficients (B), a lower number of SNS is significantly associated with higher GPA for women but not for men. Number of organizational memberships has a significant association with GPA for both men and women. As further suggested by unstandardized coefficients, number of organizational memberships, number of SNS, and GPA are all statistically significant predictors of GSR for both groups, albeit the strengths of their associations vary between men and women. Among men, the strongest predictor is number of organizational memberships followed by GPA, and number of SNS. Among women, the strongest predictor is GPA, followed by number of organizational memberships, and number of SNS. As implied by the Comparative Fit Index (CFI) and Root Mean Square Error of Approximation (RMSEA), the model with GPA as the mediator fit the data.

**Table 3:** SEM Coefficients for the Structural Relationships Between Assessed Variables Among Men and Women

Variables	Men (*N* = 1495)		Women (*N* = 1678)	
	B	ß	B	ß
Grade-point average				
Number of social networking sites	-0.003	-0.01	-0.04*	-0.08*
Number of organizational memberships	0.10***	0.12***	0.13***	0.16***
Global social responsibility				
Grade-point average	0.74**	0.06*	1.10***	0.09***
Number of social networking sites	0.68***	0.11***	0.47**	0.08**
Number of organizational memberships	1.04***	0.11***	0.51**	0.07**
*R^2^*grade-point average	.02*		.03*	
*R^2^*global social responsibility	.03*		.02*	
CFI	1.00		1.00	
RMSEA	.00		.00	

*Note.* B = Unstandardized Coefficients. ß = Standardized Coefficients. CFI = Comparative Fit Index. RMSEA = Root Mean Square Error of Approximation.

*p < .05. **p < .01. ***p < .001.

The effects of GPA, number of SNS, and number of organizational memberships on GSR were decomposed and the results are shown in Table 4. The exogenous factors have statistically significant direct and/or total positive effects on GSR, whereby increases in these factors lead to increases in GSR scores. Although the indirect effects of organizational memberships on GSR through GPA are statistically significantly robust, the indirect effects of SNS on GSR through GPA are negligible. Men and women significantly differ only with respect to the total effects of their organizational memberships on their GSR. In particular, men’s GSR is more strongly influenced by organizational memberships than women’s GSR (*p* < 0.01). Overall, gender variations in the effects of the exogenous factors are sparse, based on the p-values of the invariant constraint on the associated path coefficients shown in the last column of Table 4.

**Table 4:** Gender Variations in the Effects of Number of Social Networking Sites, Number of Organizational Memberships, and Grade-Point Average on Global Social Responsibility Scale

Variables	Men (*N* = 1495)	Women (*N* = 1678)	Group invariance in parameters
	B	B	*p*
Direct effects			
Number of social networking sites	0.68***	0.47**	0.17
Number of organizational memberships	1.04***	0.51**	0.83
Grade-point average	0.74**	1.10***	0.88
Indirect effects			
Number of social networking sites via grade-point average	-0.00	-0.04*	0.05
Number of organizational memberships via grade-point average	0.10**	0.13***	0.40
**Total effects**			
Number of social networking sites	0.67***	0.43**	0.10
Number of organizational memberships	1.14***	0.64**	0.01

*p < .05. **p < .01. ***p < .001.

## Discussion

The study examined gender variations in the effects of number of organizational memberships, number of SNS and GPA on GSR. The three factors have significantly increased GSR scores among the university students interviewed. As a mediator, GPA also has significant positive effects as hypothesized, but these effects are only true insofar as the relationships of organizational memberships and GSR are concerned. Overall, men’s GSR can be significantly differentiated from that of women’s solely in terms of organizational memberships.

The lack of broad gender variations may be accounted for by the homogeneous university life experiences, similarities in parental education, or common socio-demographic conditions among the university student population surveyed. For example, most students were young (mean age: 17.9) and freshmen who were completing general education rather than major subjects at the time of the survey, and were from high-income families. Moreover, the lack of appreciable gender differences, which could have impacted on the relational outcomes of the variables examined, may be due to a narrowing of the gender gap in the emotional expressiveness among the younger generation. The increasing numbers of young men coming to terms with their emotional side may mean shifts in the ways they will value and learn from their social and academic lives, which have implications for their GSR.

The exogenous factors are highly effective most probably because the resultant outcomes of their provisions to university students are closely related to the elements of GSR. Multiple organizational memberships help broaden young people’s sense of community service and volunteerism (Dugan & Komives, 2007; Wolfe, 2014). Multiple SNS enable young people to establish and develop cross-cultural interactions and relationships in virtual networks (Ellison et al., 2009; Lee et al., 2015). As a robust indicator of academic fortitude and broad-based learning, GPA hastens young people’s global readiness, including their global awareness and GSR (Lee et al., 2015).

Of the three factors, organizational memberships are the most significant predictor of young men’s GSR. The overall significance of organizational memberships in men may be linked to their learning process in organizations, which is mainly experiential and action-based. Being activity-oriented themselves (Cleveland, Stockdale, & Murphy, 2000), young men may be prone to learning more when the process is attuned to their learning style. In contrast, young women—with their propensities to be expressive (Noller, 1986) and deep when discussing issues (Cleveland et al., 2000), which are effective for learning social issues, may gravitate more toward classroom-based activities to acquire an in-depth knowledge of concepts such as GSR. The lack of gender variations in the effects of SNS may be due to the possibility that the men and women interviewed are still in a formative phase of exploring the higher-level functions and benefits of the Internet. Similarly, the absence of gender differences in the effects of GPA could be because the students who were interviewed, being mostly freshmen, have only been given sparse information on GSR.

The lack of appreciable mediating effects of GPA on SNS-GSR nexus may be attributed to an absence of the intertwining between GPA and SNS in terms of their respective functions. To date, GPA, which is largely academic and intellectual, and SNS, which is largely personal and social, remain as two distinct and separate domains in the Philippines. A fusion of the two, which would transform SNS into a vital academic learning tool that young people could use to deepen their learning of social issues, is a rare university agenda. The significantly strong mediating effects of GPA on the relationships between organizational memberships and GSR could be attributed to the fact that GPA and organizational memberships are inter-related (Broh, 2002; Cooper, Valentine, Nye, & Lindsay, 1999; Darling, Caldwell, & Smith, 2005). Organizational memberships enable young people to pursue their academic interests outside of the classroom, which in turn translates into school engagement and academic achievement (Ferris et al., 2013).

### Limitations and Implications for Future Research

The limitations of this study should be noted. The participants, who were from upper-income brackets, may not be representative of the entire university student population in the Philippines. In addition, some correlation and beta coefficients obtained in the analyses are modest. This suggests that other variables might have greater effects on GSR in Filipino students than those discussed in this report. For instance, international travels and overseas education (Gordon, 2014), which are vital components of the curricular activities in global citizenship education, may be an important consideration in future studies. Moreover, the explanations used to elucidate the effects of the exogenous factors on the exogenous outcome were inferred from related literature. Future research needs to examine not just the numbers related to organizational memberships, SNS and GPA but also their substantive and contextual aspects, such as the motivations for joining organizations and specific organizational activities, the purpose for using SNS, and the content coverage of academic subjects from which aspects of GSR are learned. These substantive details would be particularly useful for a more meaningful analysis of the effects of the exogenous factors. Furthermore, the gender-based analysis would be better understood if these were based on reported gender roles among young people. Prospective studies would need to conduct an inventory of gender roles and use the data to further elucidate on gender variations if any. Finally, since this study is correlational, it could not ascertain causal relationships between the exogenous and endogenous factors.

### Conclusion

The findings have implications for university-based research and education on GSR involving young people. Multiple organizational memberships and SNS, and GPA lead to higher GSR scores. Overall, organizational memberships are a strong predictor of GSR and may be used as a main pathway to effectively foster GSR in university students. In the context of the Philippines, the path model presented here is important as it is a pioneering attempt at exploring the predictors of GSR in local young people.
